# 自体肺移植治疗中央型非小细胞肺癌的疗效

**DOI:** 10.3779/j.issn.1009-3419.2020.103.12

**Published:** 2020-08-20

**Authors:** 益俊 莫, 丽娜 林, 志新 李, 承华 钟, 峻 颜, 军 况, 国雄 杨, 建华 张

**Affiliations:** 1 518101 深圳, 南方医科大学深圳医院胸外科, 医学影像中心 Department of Thoracic Surgery, Shenzhen Hospital, Southern Medical University, Shenzhen 518101, China; 2 510520 广州, 中山大学新华学院护理学院 School of Nursing, Xinhua College of Sun Yat-Sen University, Guangzhou 510520, China; 3 200433 上海, 同济大学附属上海市肺科医院胸外科 Department of Thoracic Surgery, Shanghai Pulmonary Hospital, Tongji University, Shanghai 200433, China

**Keywords:** 自体肺移植, 肺肿瘤, 袖状切除, 全肺切除, Lung autotransplantation, Lung neoplasms, Sleeve lobectomy, Pneumonectomy

## Abstract

**背景与目的:**

全肺切除术和袖状切除术是治疗中央型非小细胞肺癌的常规术式, 但存在肺功能差不能耐受全肺切除或因为肿瘤广泛侵犯支气管和肺动脉而不能进行袖状切除的情况。本研究旨在探讨自体肺移植治疗中央型非小细胞肺癌的可行性。

**方法:**

回顾性分析2016年12月-2018年12月3例行自体肺移植的中央型非小细胞肺癌患者的诊疗资料。1例双袖状切除左肺上叶, 吻合支气管, 因肺动脉切除过长, 肺动脉吻合口张力大, 遂切断下肺静脉, 将下肺上移后再吻合肺动脉, 最后将下肺静脉与上肺静脉残端吻合。2例行左全肺切除, 体外切除左肺上叶, 将修剪、灌洗后的左肺下叶重置胸腔, 依次吻合支气管、肺动脉、肺静脉。

**结果:**

平均手术时间333 min, 平均血流阻断65 min, 平均出血量450 mL, 平均住院日18.7 d; 围手术期出现痰栓堵塞支气管1例, 经支纤镜吸痰后好转; 平均随访时间20个月, 癌症死亡1例, 术后吻合口复发及脑转移1例, 4R淋巴结转移1例(经化疗后病情稳定), 无复发生存1例。

**结论:**

对于肿瘤侵犯广泛, 不能进行袖状切除或无法耐受全肺切除的中央型非小细胞肺癌患者, 自体肺移植可在彻底切除肿瘤基础上最大限度保留肺功能, 提高术后生存质量。

## Abstract (en)

**Background and objective:**

Pneumonectomy and sleeve resection are routine operations for the treatment of central non-small cell lung cancer (NSCLC), but some patients suffered of central NSCLC, whose pulmonary function is too poor to tolerate pneumonectomy, or the tumor involves the bronchus and pulmonary artery extensively, it is hard to perform bronchovascular sleeve lobectomy. The aim of this study is to assess the feasibility of lung autotransplantation in the treatment of central NSCLC.

**Methods:**

The clinical data of 3 cases with central NSCLC treated by lung autotransplantation was reviewed from December 2016 to December 2018. One patient underwent double sleeve resection of left upper lobe with end-to-end anastomosis of the bronchus. Because the resection of the pulmonary artery was too long to perfrom a tension-free anastomosis, the inferior pulmonary vein was cut off, then the left lower lobe was moved up for an anastomosis of the inferior pulmonary vein and the stump of the superior pulmonary vein. In the other 2 cases, left pneumonectomy was performed directly, and the upper left lobe was excised *in vitro*. The lower left lobe was reset to the chest after trimming and flushing and then the bronchus, pulmonary artery and pulmonary vein were anastomosed in turn.

**Results:**

The average operation time was 333 min, the average time of vascular occlusion was 86 min, the average blood loss was 450 mL, and the average hospital stay was 18.7 d; Perioperative complications included a case of bronchial obstruction, which improved after sputum aspiration through bronchofibroscope. The average follow-up period was 20 mon; One case died of cancer, one case had recurrence of anastomotic stoma and brain metastasis, one case had 4R lymph node metastasis (stable condition after chemotherapy), and one case survived without recurrence.

**Conclusion:**

For patients with central NSCLC with extensive tumor invasion, thus inability to tolerate sleeve resection or pneumonectomy, autologous lung transplantation can preserve lung function to the greatest extent with a complete tumor resection and improve postoperative quality of life.

全肺切除术是胸外科治疗中央型肺癌的常规术式, 但因其手术并发症和死亡率高, 部分患者因肺功能差不能耐受全肺切除而丧失手术机会。随着胸外科技术的发展, 支气管和肺动脉袖状切除术^[1, 2]^已在治疗中央型肺癌上得到广泛的应用, 患者肺功能得到保留, 生活质量提高, 已经逐渐成为全肺切除术的可替代术式^[3]^。袖状切除解决了肺功能差不能耐受全肺切除的限制, 但部分患者因肿瘤侵犯支气管、肺动脉范围大, 需要切除的支气管、肺动脉长, 导致不能拉拢吻合而不能完成双袖状切除, 我们在临床实践中发现这类患者可以进行自体肺移植^[4, 5]^, 手术过程概括为全肺切除, 取出体外进行袖状切除, 吻合支气管、肺动脉, 将下肺静脉吻合于上肺静脉残端。

## 1 资料与方法

### 1.1 袖状切除后自体肺移植1例

患者, 男, 53岁, 左肺上叶中央型肺癌, 术前胸部强化计算机断层扫描(computed tomography, CT)检查见左主支气管堵塞, 左肺上叶肺不张, 肺实变, 左侧胸腔缩小(图 1A), 肿瘤61 mm×53 mm×65 mm, 肿瘤包绕左肺动脉干(图 1B、图 1C), 三维重建显示左主支气管折断(图 1D), 支气管镜检查见左肺上叶支气管开口菜花状肿物, 活检为鳞癌, 术前肺功能检查第一秒用力呼气容积(forced expiratory volume in one second, FEV_1_)0.96 L, 极度阻塞性通气功能障碍, 动脉血氧分压(partial pressure of artery, PaO_2_)57.7 mmHg, 血氧饱和度(saturation oxygen, SO_2_)91.8%, 术前诊断左肺上叶中央型鳞癌cT4N0M0 IIIa期。2016年12月12日开胸探查, 见左肺上叶肺不张, 肺实变, 肺门呈冻结状, 肿瘤侵犯肺动脉干, 实施左肺上叶双袖状切除, 在主动脉弓下方切开纵隔胸膜, 显露左肺动脉主干, 1 mg/kg肝素进行全身系统性抗凝, Romel在动脉韧带上缘阻断肺动脉近心端并切断之, 分离、切断上肺静脉, 因肺动脉下切缘分离困难, Romel在近心房处阻断下肺静脉, 打开斜裂, 在下肺背段动脉上方0.5 cm处切断肺动脉远心端, 在左肺上叶支气管开口上缘切断左主支气管, 在下肺支气管开口上0.5 cm处切断支气管, 将左肺上叶取出, 4-0 PDS吻合支气管, 由于肺动脉切除长, 肺动脉吻合口张力大, 遂用吻合器切断下肺静脉, 将下肺上移, 肝素盐水冲洗血管残端, 5-0 Prolene吻合肺动脉, 切除下肺静脉吻合钉, 用4-0 Prolene将下肺静脉与上肺静脉残端进行吻合, 血管吻合最后一针暂不结扎, 松开Romel待血液流出排气后再收紧最后一针缝线。患者为姑息性切除, 未清扫纵隔淋巴结。手术时间300 min, 血流阻断30 min, 术中出血600 mL, 输红细胞2 U。术后在重症加强护理病房(intensive care unit, ICU)用呼吸机辅助呼吸2 d。术后第4天出现呼吸困难, 支气管镜检查发现痰栓堵塞术侧支气管, 经吸痰后症状好转, 第14天康复出院, 出院时血气分析PaO_2_为66.2 mmHg, SO_2_为93.9%, 氧分压、氧饱和度较术前改善, 术后病理为中分化鳞癌, 支气管残端无癌组织残留。术后DP方案(多西他赛+顺铂)辅助化疗6个周期, 术后13个月患者出现咯血, 胸部CT检查见吻合口周围肿瘤复发, 行放疗, 术后23个月磁共振检查发现脑转移, 术后总生存为24个月。

**1:**
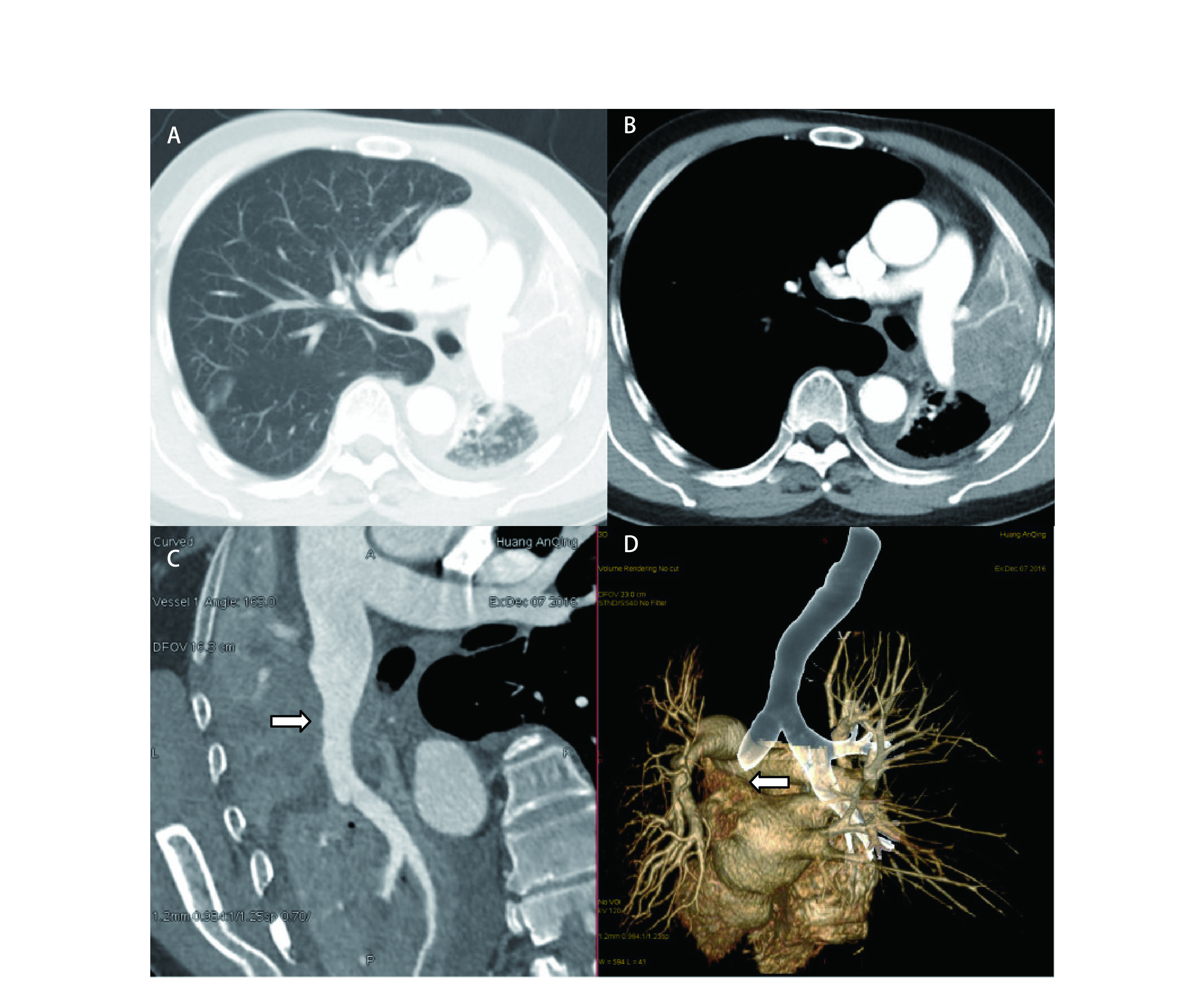
胸部增强CT检查。A：肺窗, 左主支气管堵塞, 左肺上叶肺不张, 肺实变, 左侧胸腔缩小; B：纵隔窗, 肿瘤包绕左肺动脉干, 肿瘤61 mm×53 mm×65 mm; C：矢状面重建显示肿瘤侵犯肺动脉达斜裂水平(白色箭头所示); D：三维重建显示左主支气管折断(白色箭头所示)。 Thoracic enhanced CT scanning. A: Pulmonary window: The left upper lobe atelectasis and consolidation, the left thoracic cavity reduction, because the left main bronchus blocked by tumor; B: Mediastinal window: The tumor surrounds the left pulmonary trunk. The tumor size is 53 mm×61 mm×65 mm; C: The sagittal reconstruction of the lung showed that the tumor invades the left pulmonary artery (as indicated by the white arrow); D: CT 3D reconstruction shows the left main bronchus broken (as indicated by the white arrow). CT: computed tomography.

### 1.2 全肺切除后自体肺移植2例

病例1, 男, 53岁, 左肺上叶中央型肺癌, 术前强化CT检查显示肿瘤包绕左肺动脉干(图 2A、图 2B), 肿瘤43 mm×37 mm×25 mm, 支气管镜检查见肿瘤位于左肺固有上叶, 堵塞管腔, 活检为鳞癌。予DP方案化疗2个周期, 疗效部分缓解(partial response, PR), 术前诊断左肺上叶中央型鳞癌cT4N0M0 IIIa期。2018年8月13日开胸探查, 左肺门固定, 肿瘤侵犯肺动脉达叶间裂, 需要切除的肺动脉长, 切除后不能拉拢吻合, 遂先实施左全肺切除再进行左肺下叶自体肺移植, Romel分别阻断肺动脉干及上肺静脉后切断之, 用吻合器切断下肺静脉, 在左肺上叶支气管开口上缘切断左主支气管, 将左全肺取出体外并置于4 ℃生理盐水中, 在左肺下叶背段动脉上方0.5 cm处切断肺动脉, 在下肺支气管开口上方0.5 cm处切断支气管, 对左肺下叶支气管进行插管, 麻醉呼吸机进行辅助通气, 潮气量200 mL, 生理盐水对肺血管进行顺行灌洗, 直到从肺静脉流出来的液体变清, 同时修剪支气管、肺动静脉切缘, 然后将左肺下叶重置胸腔进行自体肺移植, 在术野周围放置单层冰进行保护, 依次吻合支气管、肺动脉、肺静脉(下肺静脉与上肺静脉残端吻合), 手术时间415 min, 血流阻断97 min, 术中出血300 mL。术后病理为中-低分化鳞状细胞癌, 侵犯支气管及周围软骨, 支气管残端无癌组织残留, 第5组淋巴结无癌转移(0/3), 术后第21天步行出院, 此后继续予DP方案化疗4个周期, 随访18个月, 肿瘤无复发转移, 生活状态良好。

**2:**
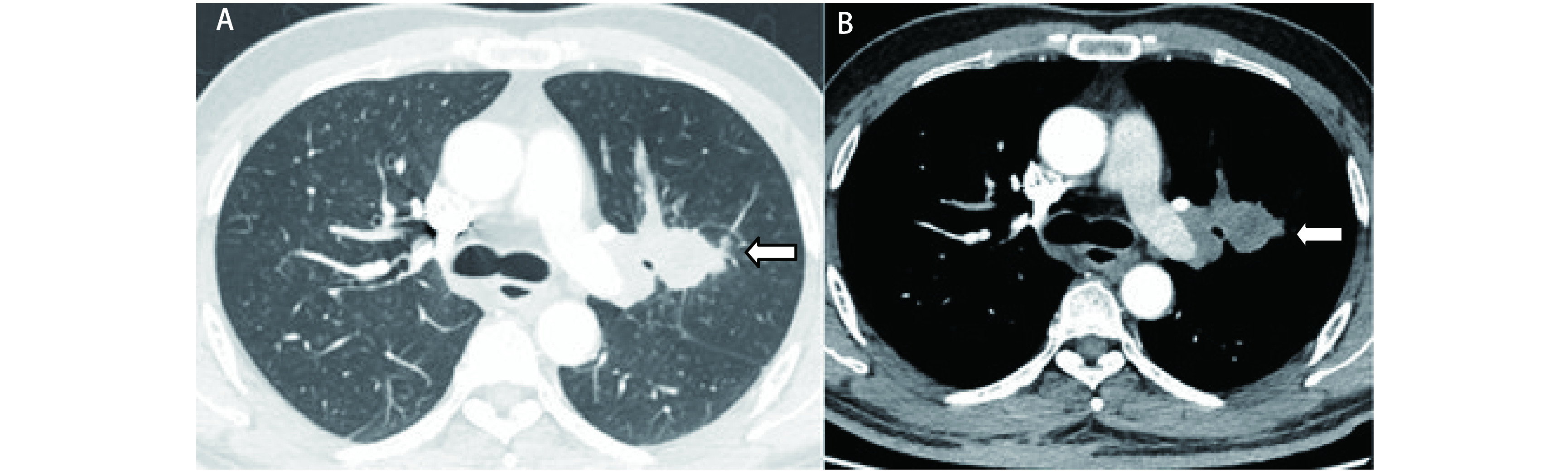
胸部增强CT检查。A：肺窗, 左肺上叶中央型肺癌, 肿瘤包绕肺动脉干(白色箭头所示); B：纵隔窗, 肿瘤呈分叶状, 包绕肺动脉干, 肿瘤43 mm×37 mm×25 mm(白色箭头所示)。 Thoracic enhanced CT scanning. A: pulmonary window: The tumor is located in the center of the left upper lobe (as indicated by the white arrow); B: mediastinal window: The tumor was lobulated and invades the the left pulmonary artery (as indicated by the white arrow). The tumor size is 43 mm×37 mm×25 mm.

病例2, 男, 63岁, 左肺上叶中央型肺癌, 术前强化CT检查显示肿瘤侵犯左肺动脉干(图 3A、图 3B), 肿瘤35 mm×21 mm×15 mm, 支气管镜检查见肿瘤堵塞左肺固有上叶支气管, 活检为鳞癌, 予GP方案(吉西他滨+顺铂)化疗1个周期, 疗效部分缓解(partial response, PR), 患者拒绝继续化疗, 迫切要求手术切除, 术前诊断左肺上叶中央型鳞癌cT4N0M0 IIIa期。2018年8月24日开胸探查, 术前规划实施左肺上叶双袖状切除, 术中分离肺动脉下切缘困难, 可能损伤下叶基底干, 另外需要切除的肺动脉长, 吻合口张力大, 根据我们以上两例自体肺移植的成功经验, 决定实施左全肺切除, 在体外分离肺动脉下切缘, 然后再移植左肺下叶, 手术过程同上例, 手术时间285 min, 血流阻断67 min, 术中出血450 mL, 术后病理为中分化鳞状细胞癌, 支气管残端无癌组织残留, 淋巴结无癌转移(4L组：0/3, 5组：0/2, 7组：0/2, 9组：0/2, 10组：0/2), 术后第21天康复出院, 术后2个月复查CT见4R组淋巴结转移, 予GP方案辅助化疗2周期, 疗效疾病进展(progressive disease, PD), 改TP方案(紫杉醇+顺铂)化疗4个周期, 疗效疾病稳定(stable disease, SD), 随访18个月, 生活质量佳。

**3:**
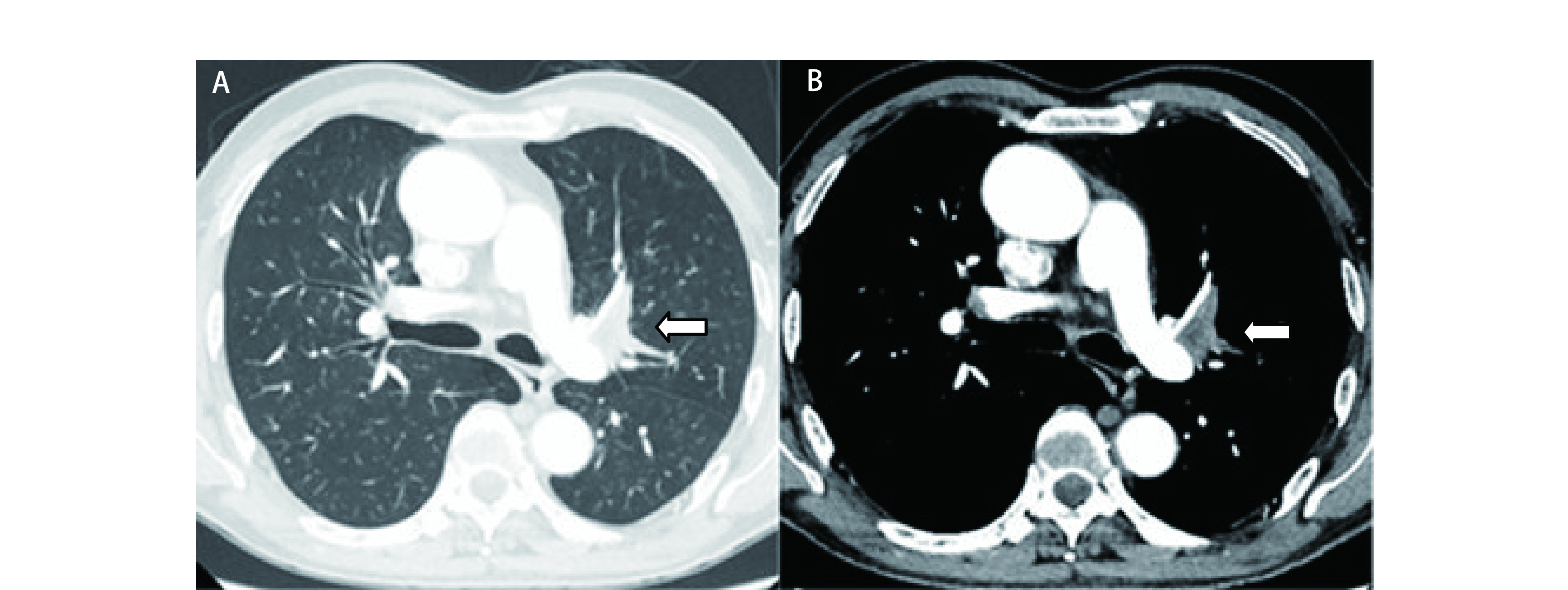
胸部增强CT检查。A：肺窗, 左肺上叶中央型肺癌(白色箭头所示); B：纵隔窗, 肿瘤侵犯肺动脉干, 肿瘤35 mm×21 mm×15 mm(白色箭头所示)。 Thoracic enhanced CT scanning. A: pulmonary window: The tumor is located in the center of the left upper lobe (as indicated by the white arrow); B: Mediastinal window: The tumor invades the the left pulmonary artery, the tumor size is 35 mm×21 mm×15 mm (as indicated by the white arrow).

### 1.3 统计分析

使用SPSS 17.0统计软件对统计结果进行分析。收集患者围手术期资料, 记录肿瘤大小、病理诊断、肿瘤原发灶-淋巴结-转移(tumor-node-metastasis, TNM)分期, 手术时间、血流阻断时间、出血量、随访时间。

## 2 结果

平均手术时间333 min, 平均血流阻断65 min, 平均出血量450 mL, 平均住院日18.7 d; 围手术期出现痰栓堵塞支气管1例, 经支纤镜吸痰后好转; 平均随访时间20个月, 癌症死亡1例, 术后吻合口复发及脑转移1例, 4R淋巴结转移1例(经化疗后病情稳定), 无复发生存1例。

## 3 讨论

肺癌是当今威胁人类健康的第一大恶性肿瘤, 手术切除是目前治疗非小细胞肺癌最有效的手段之一。对中央型肺癌, 全肺切除因并发症及死亡率高, 术后患者生活质量差, 已在临床谨慎使用, 局部进展期非小细胞肺癌是支气管、肺动脉袖状切除的明确适应证, 袖状切除可以保留肺功能, 其死亡率、生存率与肺叶切除相似^[6, 7]^, 比全肺切除安全而疗效相当^[8]^, 甚至优于全肺切除^[9]^, 目前已在临床中广泛应用^[10]^。但我们在临床中发现部分中央型肺癌侵犯范围大, 切除更长的支气管、肺动脉后不能吻合, 在这种情况下可以融合全肺切除与袖状切除两种外科技术完成手术, 手术过程概括为全肺切除, 取出体外进行袖状切除, 吻合支气管、肺动脉, 下肺静脉吻合于上肺静脉残端, 我们把这项技术称为自体肺移植^[11, 12]^。

自体肺保存温度、保存液的研究尚不明确。本研究中3例患者中2例离体肺置于4 ℃生理盐水中保存并进行肺血管灌洗, 1例未离体及灌洗, 3例均移植成功。张国良等^[11]^4例自体肺移植, 其中2例用室温肝素生理盐水(12, 500 U/500 mL)保存、灌洗, 移植成功, 而使用8 ℃肝素生理盐水的2例移植失败。Shiono等^[13]^使用20 ℃肝素生理盐水(12, 500 U/500 mL)保存6例成功, 1例失败。移植失败的原因不一定与保存液的温度有关, 但说明使用室温肝素盐水保存、灌洗移植肺是可以移植成功的。Oto等^[4]^应用磷酸盐缓冲液EP-TU保存灌洗移植肺获得成功, 部分研究^[12, 13]^显示移植前未使用溶液进行保存、灌洗亦取得移植成功。肺冷缺血的上限还不明确, 冷缺血时间 < 6 h相对安全, 在异体肺移植手术中研究发现冷缺血时间 > 6 h组原发性移植物失功发生率要高于冷缺血时间 < 6 h组^[14]^。多伦多肺移植中心^[15]^研究发现优质供肺的冷缺血时间超过12 h后移植仍然能够获得满意结果, 但对边缘供肺仍把缺血时间限制在8 h以内。这些研究表明自体肺移植从血管吻合到血液再通的时间是充足的。

吻合技术是影响自体肺移植成败的重要因素, 缝合前需要选择合适的缝线, 我们对支气管、肺动静脉进行连续吻合, 支气管吻合使用4-0 PDS, 肺动脉使用5-0 Prolene, 肺静脉使用4-0 Prolene, 血管吻合完成即可建立血液循环, 但支气管吻合在术后仍有发生支气管胸膜瘘可能, 张国良等^[11]^研究中4例自体肺移植, 其中2例失败均与支气管胸膜瘘有关, 支气管胸膜瘘是严重的术后并发症, 一旦发生需要切除移植肺, 故支气管吻合要更为细致, 缝合的间距、厚度合理, 缝合完毕用神经拉钩逐条收紧缝线, 膨肺试漏, 吻合口应能耐受30 mmH_2_O的压力而不漏气, 否则需要加固缝合, 对支气管近端、远端直径差别较大的, 可以采用望远镜式支气管吻合法^[16]^。支气管、血管吻合完毕可以用带蒂的肋间肌、胸膜组织、主动脉外膜、大网膜^[14]^包绕在吻合口周围。

我们在离断肺动脉前使用肝素进行系统性抗凝, 术后未抗凝治疗, 没有患者出现肺栓塞, 但术中或术后是可能产生血栓的, 多项研究^[12, 13]^在术前使用肝素(1 mg/kg)进行系统性抗凝, 术后肺静脉栓塞的主要原因可能是离体肺血管灌洗不当、静脉吻合口狭窄、血管扭曲或受压、抗凝不充分、离体肺再灌注损伤等^[11]^。张国良等^[11]^术后应用肝素50 mg/24 h, 连续5 d, Filosso等^[15]^在术后抗凝治疗1个月, Shiono等^[13]^在术后使用低分子肝素钙2, 850 IU/d, 连续7 d。综上所述, 建议自体肺移植术前术后进行充分的抗凝治疗, 术后抗凝治疗需警惕出血风险, 监测凝血功能。

移植肺在体外插管, 呼吸机辅助通气, 可以使塌陷的肺泡重新复张, 肺的舒缩运动有助于灌洗液将肺内的残血彻底清除。术后保留气管插管, 在ICU使用呼吸机辅助呼吸有助于预防术后肺不张, 结合使用支气管镜对加强呼吸道的管理有重要作用。

支气管镜在自体肺移植术前、术后的使用具有重要的意义。术前支气管镜检查可以取活检明确诊断, 评估切除范围, 术后可以直观了解吻合口愈合情况, 还可以清除气道分泌物、血凝块, 解除呼吸道梗阻, 避免肺不张及肺部感染的发生, 取深部痰进行细菌培养及药敏试验, 为抗生素使用提供依据。支气管镜检查在喉罩全麻下进行可以减轻患者痛苦。

系统性淋巴结清扫可以了解肺癌淋巴结转移情况, 对于明确肺癌的分期、指导术后综合治疗以及判断预后都有着重要意义, 未进行系统性淋巴结清扫是本研究存在的不足之处。另外, 本研究样本量小, 自体肺移植的并发症、疗效等还需要进一步的探讨。

综上所述, 自体肺移植适用于肿瘤侵犯广泛, 不能进行双袖切除或不能耐受全肺切除的中央型非小细胞肺癌患者。自体肺移植技术的优点：①在完全切除肿瘤基础上能最大限度地保留肺功能, 提高术后生存质量; ②体外切除肿瘤使手术变得更从容, 减少术中出血量及缩短手术时间; ③冷保存移植肺及生理盐水灌洗条件容易获得; ④不需要人工材料; ⑤自体肺移植手术过程与异体肺移植相似, 在自体肺移植积累的经验有助于开展异体肺移植手术。
